# PlantIntronDB: a database for plant introns that host functional elements

**DOI:** 10.1093/database/baad082

**Published:** 2023-11-09

**Authors:** Weiping Wang, Jiming Hu, Han Li, Jun Yan, Xiaoyong Sun

**Affiliations:** Agricultural Big Data Research Center, College of Information Science and Engineering, Shandong Agricultural University, Taian, Shandong 271018, China; Agricultural Big Data Research Center, College of Information Science and Engineering, Shandong Agricultural University, Taian, Shandong 271018, China; Agricultural Big Data Research Center, College of Information Science and Engineering, Shandong Agricultural University, Taian, Shandong 271018, China; Agricultural Big Data Research Center, College of Information Science and Engineering, Shandong Agricultural University, Taian, Shandong 271018, China

## Abstract

Although more and more attention has been focused on introns and the important role of plant introns in plant growth and development has been discovered, there is still a lack of an open and comprehensive database on plant introns with functional elements in current research. In order to make full use of large-scale sequencing data and help researchers in related fields to achieve high-throughput functional verification of identified plant introns with functional elements, we designed a database containing five plant species, PlantIntronDB and systematically analyzed 358, 59, 185, 210 and 141 RNA-seq samples from *Arabidopsis thaliana (Arabidopsis), Gossypium raimondii* (cotton), *Zea mays* (maize), *Brassica napus* (oilseed rape) and *Oryza sativa Japonica Group* (rice). In total, we found 100 126 introns that host functional elements in these five species. Specifically, we found that among all species, the number of introns with functional elements on the positive and negative strands is similar, with a length mostly smaller than 1500 bp, and the Adenine/Thymine (A/T) content is much higher than that of Guanine/Cytosine (G/C). In addition, the distribution of functional elements in introns varies among different species. All the above data can be downloaded for free in this database. This database provides a concise, comprehensive and user-friendly web interface, allowing users to easily retrieve target data based on their needs, using relevant organizational options. The database operation is simple and convenient, aiming to provide strong data support for researchers in related fields to study plant introns that host functional elements, including circular RNAs, lncRNAs, etc.

**Database URL:**  http://deepbiology.cn/PlantIntronDB/

## Introduction

Introns are non-coding nucleotide sequences that do not appear in mature mRNAs (1). Central dogma states that DNA encodes information about genetic material that is first transcribed into RNA and then translated into proteins (2). Transcription and translation constitute the entire process of gene expression, and RNA splicing is a crucial step in this process (3). During splicing, introns in pre-mRNA are removed and exons are joined in an orderly manner, which are finally processed into mature mRNA for translation, and then, introns are rapidly degraded (4, 5). Therefore, introns have always been considered as dispensable by-products and have been neglected for a long time (6).

However, in recent years, several studies have revealed biological significance of introns and found that introns play an indispensable regulatory role (7–9). For example, two studies in 2019 reported that in yeast cells, one or more strains lacking the removed introns had more difficulty surviving under nutrient-poor conditions than wild-type yeast, demonstrating the importance of introns under nutrient-poor conditions and suggesting that introns are necessary for the survival of cells in nutrient deprivation (10, 11). In addition, introns with functional elements have been found to play an important role in regulating biological processes in tropical *Xenopus oocytes* (12–14), human cell lines (15–19), Drosophila (20–23), *Arabidopsis* (24, 25) and other organisms (26–28). What’s more, in our recent study, we designed Intron-capture RNA-seq to study introns that host functional elements in *Arabidopsis* (29). Despite the increasing attention focused on introns, it seems that a comprehensive database on introns that host functional elements has not yet emerged as far as the current studies are concerned. Plant Intron-Splicing Efficiency Database focuses only splicing of introns, which may not reveal the detailed landscape of functional elements in introns (30).

In this study, we developed a user-friendly database of plant introns containing functional elements: PlantIntronDB. This database currently contains a total of 100 126 introns with functional elements, including 19 363 in *Arabidopsis*, 2334 in cotton, 13 079 in maize, 31 370 in oilseed rape and 33 980 in rice, which can be downloaded for free. We found that the introns with functional elements in all species have similar numbers of positive and negative strands, with lengths mostly less than 1500 bp, and the content of A/T is much higher than that of G/C. In addition, we also found that the distribution of intron functional elements in different species has different characteristics. To the best of our knowledge, this is the first database currently available for the study of introns with functional components in plants. The database provides a clean and comprehensive, easy-to-use web interface so that users can easily search targets, using gene ID, chromosome, intron number, width, start, end, strand and species. The database is easy to operate and detailed information on all plant introns can be displayed. Placing these data on a unified browsing platform should allow high-throughput functional validation of identified plant introns and facilitate community studies on the regulatory roles of plant introns hosting functional elements.

## Materials and methods

### All plant data analysis pipeline

We selected five plant species, including *Arabidopsis*, cotton, maize, oilseed rape and rice, and searched the NCBI SRA database (https://www.ncbi.nlm.nih.gov/sra) for related RNA-seq samples on 8 July 2022. We used advanced search terms, including ‘RNA-seq’ and ‘Illumina’, and downloaded RNA-seq data for each species. Then, we used the SRAToolkit v3.0.2, Hisat2 v2.2.1 (31), and other software packages (details in 2.2) to process and analyze the data. We deleted files that did not generate valid sequence data during processing and then selected specific element features (geneID, intronNo, chr, strand, start, end, width, sampleNumber) to be entered into our database. Due to the fact that using more samples to process data can achieve better data purity, subsequent research will expand the sample size of the database to contain more species, which is also a suitable method to improve the reliability and comprehensiveness of the database. At the same time, the plant data we processed will be publicly released on our website. (http://deepbiology.cn/PlantIntronDB/).

### Detection of introns hosting functional elements

In our study, we processed RNA-seq data and aligned the clean reads to the reference genomes of *Arabidopsis thaliana (Arabidopsis)* (TAIR10), *Gossypium raimondii* (cotton) (Graimondii2_0_v6), *Zea mays* (maize) (Zm-B73-REFERENCE-NAM-5.0), *Brassica napus* (oilseed rape) (AST_PRJEB5043_V1) and *Oryza sativa Japonica Group* (rice) (IRGSP-1.0) by using Hisat2. After alignment, we used Samtools v1.9 (32) to sort and index the bam files for subsequent analysis. Then, we used the Bioconductor packages [GenomicRanges v1.32.6 (33), GenomicAlignments v1.16.0 (33) and Biostrings v2.48.0 (34)] to analyze the bam files. Specifically, we compared the sequencing reads to the genome annotation by using the ‘countOverlap’, ‘findOverlap’ and ‘subsetByOverlap’ functions from the GenomicRanges and GenomicAlignments packages. We also used ‘translateGTF’ function from SplicingTypesAnno v1.0.2 (35) to input gene annotation files in GFF/GTF format to extract detailed features from introns, such as gene ID, start and end position, strand and intron number. We used the ‘DNAStringSet’ and ‘substring’ from the Biostrings package to extract nucleotide sequences. Finally, we used R packages [ggplot2 v3.3.5 (36) and lattice v0.20–38 (37)] for visualization.

To ensure the quality of the introns hosting functional elements, we followed our previous work (29) to process the reads. Specifically, when selecting reads, we followed the following criteria: (i) reads are inside introns, (ii) reads are strand-specific as introns and (iii) reads have no junctions. Besides, we removed all reads that overlapped both exons and introns. Then, all the reads within the introns were merged using the ‘reduce’ function. When identifying introns hosting functional elements, we selected candidates with the following criteria: (i) > 90% read coverage in the intron and (ii) found in at least seven mRNA-seq samples. We used the IGV (Integrative Genomics Viewer) v2.11.9 (38) to ensure the reliability of the selected data.

### Database development

We developed a database to store plant intron data information, using PHP and MySQL. The data in this database not only include gene sequences but also essential information, such as species, gene ID, intron number, chromosome, strand, start and end position, width and sample number. Our data are limited to hundreds of RNA-seq samples from NCBI SRA for five species. However, as the amount of data increases, more than a single-layer MySQL database may be required to handle the data storage and retrieval requirements. Therefore, our database technology will shift towards distributed databases to better handle the challenges of storing massive amounts of data.

## Results and discussion

### Introns hosting functional elements for five plant species

**Figure 1.**
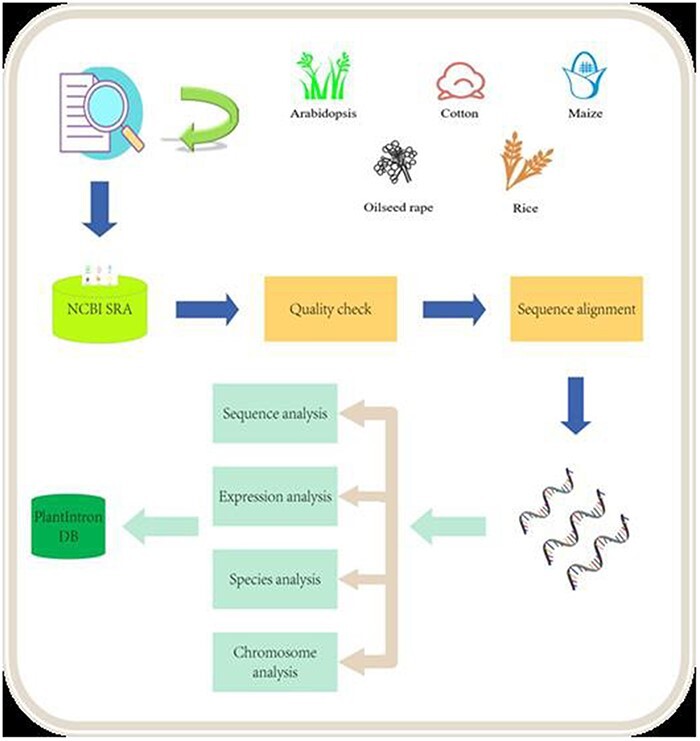
Data analysis pipeline. The raw data from NCB SRA were downloaded and processed after quality check. The alignment data were then analyzed for identifying functional introns. The final results were deposited into the PlantIntronDB.

In this study, we downloaded the data from NCBI SRA database and analyzed 358 samples of *Arabidopsis*, 59 samples of cotton, 185 samples of maize, 210 samples of oilseed rape and 141 samples of rice, respectively (details are available on the website). We used SRAToolkit, Hisat2 and other software packages to analyze the above RNA-seq samples. We removed files that did not generate valid sequence data during the analysis pipeline through data cleaning and only retained files that met the standards for analysis (Figure 1). In total, 100 126 introns that host functional elements were found in *Arabidopsis*, cotton, maize, oilseed rape, and rice, including 19 363 in *Arabidopsis*, 2334 in cotton, 13 079 in maize, 31 370 in oilseed rape and 33 980 in rice (Table 1).

**Table 1.** The number of functional introns in five species

Species	Introns hosting functional elements
*Arabidopsis thaliana (Arabidopsis)*	19 363
*Gossypium raimondii* (Cotton)	2334
*Zea mays* (Maize)	13 079
*Brassica napus* (Oilseed rape)	31 370
*Oryza sativa Japonica Group* (Rice)	33 980

**Figure 2.**
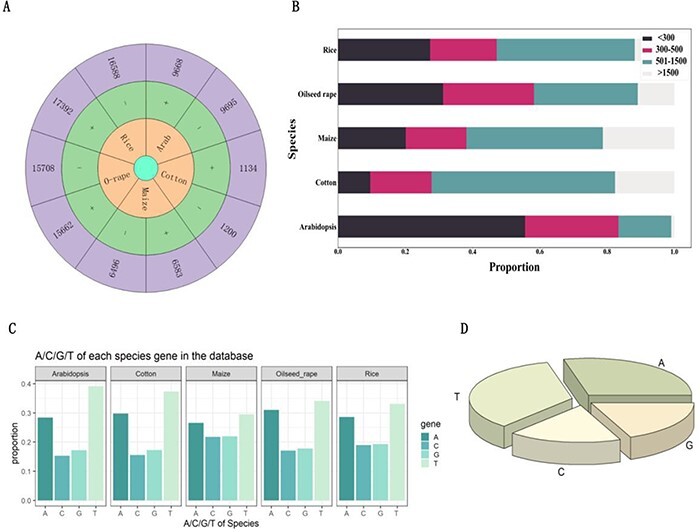
Analysis of introns that hosting functional elements. (A) Number of introns carrying functional elements on the positive and negative strands of *Arabidopsis*, cotton, maize, oilseed rape and rice. (B) Length of introns with functional elements in *Arabidopsis*, cotton, maize, oilseed rape and rice. (C) The proportion of each base in introns with functional elements of *Arabidopsis*, cotton, maize, oilseed rape and rice. (D) The proportion of bases in all introns with functional elements.

Figure 2A provides the number of introns hosting functional elements on the positive and negative strands of each species, where ‘+’ and ‘−’ represent positive and negative strands, respectively. From the figure, we found that the number of introns hosting functional elements on both the positive and negative strands of five species is similar. In addition, this study also analyzed the length of introns with functional elements. The different sequence lengths in the five species are shown in Figure 2B. It can be seen that in all species, introns with functional elements >1500 bp in length are far less than those with lengths <1500 bp, indicating that most introns with functional elements have sequence lengths within 1500 bp. Figure 2C shows the content of each base in introns with functional elements. It can be seen from the figure that the content of A and T in introns with functional elements of various species is significantly higher than that of G and C. While integrating five species, it was discovered that the proportion of A and T is much greater than that of G and C (Figure 2D). These results will help future researchers to gain a deeper understanding of the biological characteristics and functions of introns hosting functional elements.

**Figure 3.**
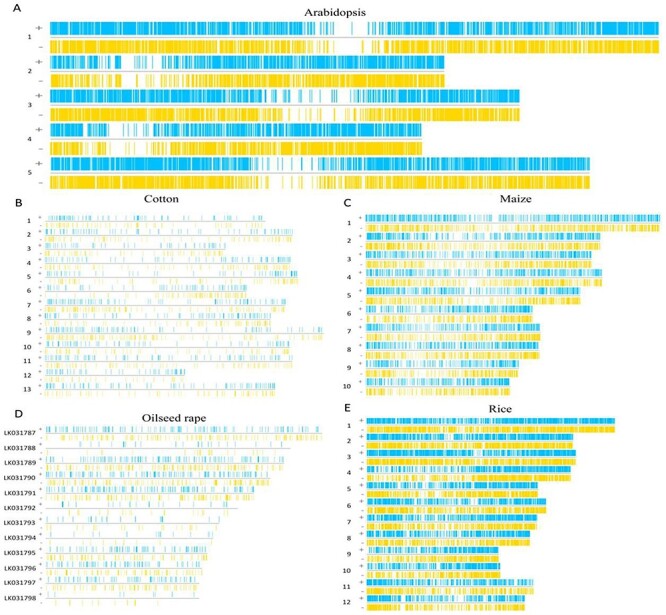
The region of functional elements in introns. (A) The functional elements of *Arabidopsis* introns are distributed on its chromosomes. (B) The functional elements of cotton introns are distributed on its chromosomes. (C) The functional elements of maize introns are distributed on its chromosomes. (D) The functional elements of oilseed rape introns are distributed on its chromosomes. (E) The functional elements of rice introns are distributed on its chromosomes.

The functional elements in the introns of five species have roughly the same distribution on the positive and negative strands of their chromosomes. Specifically, functional elements in *Arabidopsis* introns are the most widely and densely distributed (Figure 3A) and shows similar representation in maize and rice (Figure 3C and E). In cotton and oilseed rape, the distribution of functional elements in introns is quite sparse (Figure 3B and D).

### Database features

Currently, PlantIntronDB provides the following information: (i) gene ID. This ID integrates the higher-level ID to which the feature is subordinate, which helps to associate introns hosting functional elements with genes, and it enables users to compare and search different species of introns; (ii) intron-specific information for each species, including intron number, which refers to the position of the intron, chromosome, start position, end position, positive and negative strand, sequence length and sample number. The sample number is how many samples have been analyzed from all samples to contain introns with functional elements. At present, this database mainly supports the following functions: (i) intron information browsing: users can browse the specific attributes of all data in the database to understand its basic information, providing convenience for subsequent research; (ii) intron search: users can quickly locate an intron of interest by entering information such as gene ID, intron number, chromosome, start, end, width, strand and species; (iii) intron download: users can obtain complete information about all introns through the download function.

**Figure 4.**
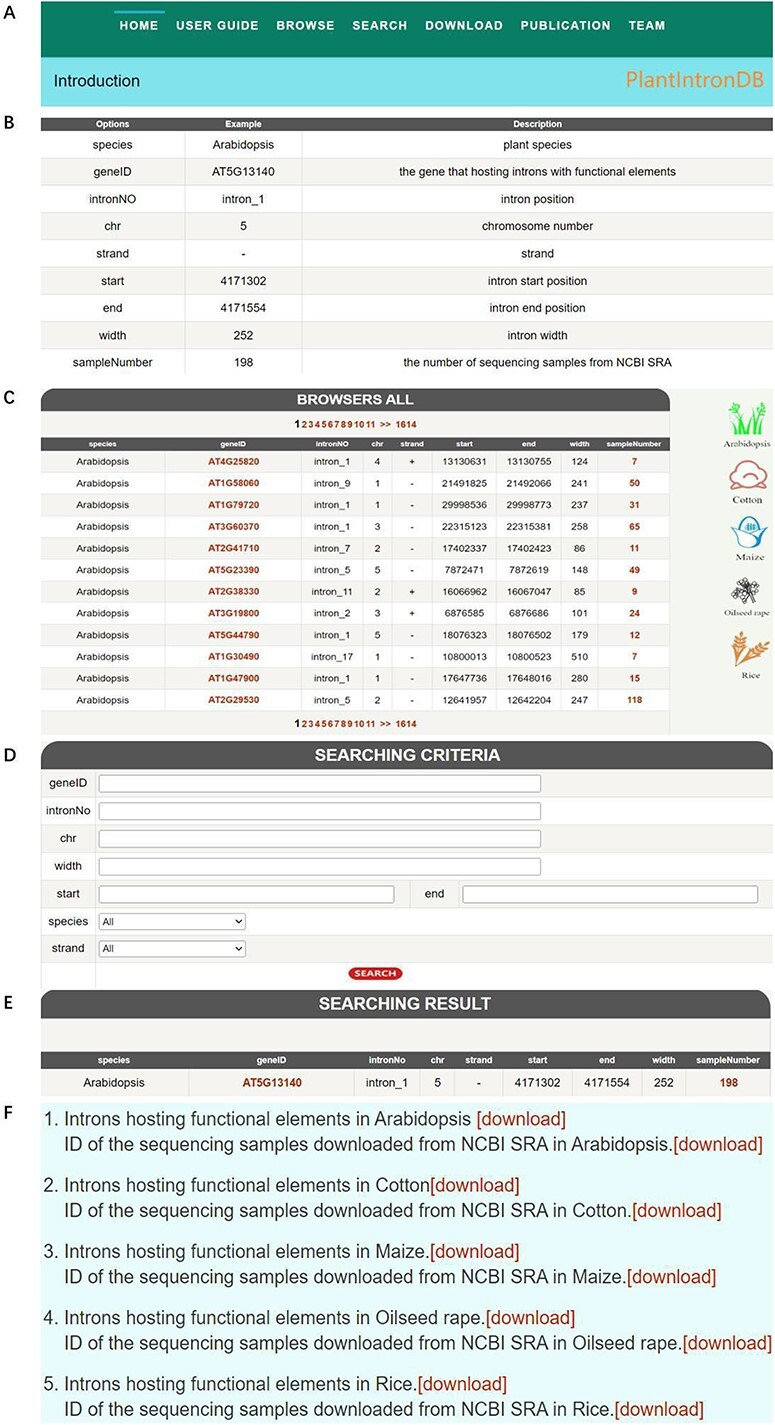
PlantIntronDB database. (A) PlantIntronDB navigation bar. (B) PlantIntronDB user guide page. (C) PlantIntronDB browsing page. (D) PlantIntronDB search page. (E) Search result of PlantIntronDB. (F) PlantIntronDB download page.

The web interface of PlantIntronDB consists of seven pages: Home, User Guide, Browse, Search, Download, Publication and Team (Figure 4A). Home introduces introns hosting functional elements and the database information. The User Guide page gives a guide to use the database and examples of searched introns hosting functional elements (Figure 4B). The Browse page provides detailed information about all data, i.e. species, gene ID, intron number, chromosome number, strand, start position, end position, sequence length and sample number. On the right side of the page, logos for five species are provided. Clicking on the logos will display detailed information on all introns hosting functional elements for the corresponding species (Figure 4C). The search portal is the main function of the website, providing eight attributes, and users can search for the target intron by entering and selecting the corresponding attribute of the search target (Figure 4D and E). The download module provides intron data and RNA-seq samples ID of five plants; users can download all data and sample IDs for free by clicking the corresponding option (Figure 4F). The Publication page shows our team’s published papers related to this research, and the Team page gives a brief introduction of our team.

## Conclusion

PlantIntronDB provides a complete and comprehensive platform for plant introns, including *Arabidopsis*, cotton, maize, oilseed rape and rice. It has detailed information about introns hosting functional elements, including gene ID, chromosome number, intron number, strand, start and end position and sample number. In addition, the platform also provides a search function to facilitate users to find the search target quickly. Finally, it also provides a free download function, which allows users to download by clicking the links of corresponding species. By using this database, users can not only grasp the rich details of plant introns hosting functional elements but also track these introns’ resources and reveal their novel functions and regulatory roles. We believe that this database will help the community to have a deeper understanding of plant introns with functional elements.

## Data Availability

All related data are available at http://www.deepbiology.cn/PlantIntronDB/.
